# "There's nothing I can't do – I just put my mind to anything and I can do it": a qualitative analysis of how children with chronic disease and their parents account for and manage physical activity

**DOI:** 10.1186/1471-2431-9-1

**Published:** 2009-01-01

**Authors:** Jennifer Fereday, Colin MacDougall, Marianne Spizzo, Philip Darbyshire, Wendy Schiller

**Affiliations:** 1 Children, Youth and Women's Health Service, 72 King William St, North Adelaide, South Australia, Australia; 2 Department of Public Health, School of Medicine, Flinders University, GPO Box 2100, Adelaide, South Australia, Australia; 3 Children, Youth and Women's Health Service, University of South Australia and Flinders University, South Australia, Australia; 4 Division of Education, Arts and Social Sciences, University of South Australia GPO Box 2471, Adelaide, South Australia, Australia

## Abstract

**Background:**

This paper reports the findings of a South Australian qualitative, exploratory study of children and young people living with a chronic disease, and their perceptions and experiences of physical activity. The perceptions and experiences of their parents were also explored. The chronic diseases were type 1 diabetes, asthma and cystic fibrosis.

**Methods:**

Multiple qualitative data collection techniques were used to elicit the children and young people's perspectives and experiences of physical activity, including focus groups, maps, photos and 'traffic light posters'. The children's parents were interviewed separately to ascertain their views of their child's participation in physical activities.

**Results:**

Children and young people described their active participation in a wide variety of physical activities including organised sports and play, but made very little mention of any negative influence or impact due to their disease. Their parents' stories described the diligent background planning and management undertaken to enable their child to participate in a wide range of physical activities.

**Conclusion:**

The results of this study suggest that for these children and young people, having a chronic disease was not perceived as a barrier to participation in organised sport and recreational activities. They were physically active and perceived themselves to be no different from their peers. Their positive beliefs were shared by their parents and the level of participation described was enabled by the high level of parental support and background planning involved in managing their child's health care needs.

## Background

Physical inactivity is a growing international child health concern [1], considered to be one of the most important risk factors for significant adult morbidity and mortality [2]. It is argued that being physically active is important for children's overall physical, emotional and social health and wellbeing and that it is a positive benefit that extends into adult life [3,4].

Children are neither a homogenous nor a universally healthy population. In Australia the prevalence of asthma has been reported as 24.8% of 0–17 year olds [5], the prevalence of type 1 diabetes among 0–14 year olds as 19.2/100,000 in boys and 18.6/100,000 in girls, and the prevalence of cystic fibrosis as 40 per 100,000 [6]. The care of children with chronic diseases can be extremely complex and the cost is high for children and their families in social, psychological and economic terms [6].

If lifetime physical activity and health patterns are established in childhood and healthy, active children seem more likely to become healthy active adults, it follows that children with type 1 diabetes, asthma or cystic fibrosis should benefit from regular physical activity as an essential part of their overall health care program [7,8]. Benefits of exercise for children with type 1 diabetes include weight control, prevention of later cardiovascular disease and the psychosocial benefit of enhanced feelings of self-worth [9,10]. Aerobic fitness for children with asthma improves their breathing reserve [8]. Although a Cochrane Collaboration Review [11] on physical training for cystic fibrosis reported inconsistency of study results in relation to efficacy, they concluded the anticipated benefits to lung function and feelings of self-esteem from engaging in physical activity were widely supported by health professionals who encourage exercise as part of an overall cystic fibrosis care package.

The child with a chronic disease at school was often 'the protected child' and encouraged to 'play piano rather than baseball' [12]. Vitulano [13] describes how participation in sport for children with a chronic disease may be more of a 'struggle' rather than a fun activity. Lang et al's study provided evidence from care-giver reports that urban school-aged children with asthma were less active than their peers. These results correlated with disease severity, however parental health beliefs also contributed to the lower activity level of children with asthma [14]. Perceptions and beliefs about exacerbation of symptoms of a chronic disease such as asthma, in the absence of physiological evidence, have been found to be a barrier to participation in physical activity for some children and young people [15]. As Sawyer noted [16] in relation to children with asthma, perceptions were often that: "students with asthma were not able to do all the things that children without asthma could do including sport and outdoor activities, that children with asthma were physically weaker than those without" (p 483).

Participation in physical activity is an integral part of a child's development [13] and recent initiatives such as the 'Charter of Physical Activity and Sport for Children and Youth' [17] emphasise that access and opportunity to participate in physical activity should be made available to all, including children with disabilities and chronic illness. However, access and opportunity factors alone will have little effect on improving physical activity patterns unless clearer understandings are developed of how these children and young people view, understand and experience play, sport, exercise and physical activity within the context of their worlds as children living with an ongoing health problem. Very few studies have explored this area of children's perspectives and these tend to focus on adolescents. Williams [18] examined the gender differences in chronically ill boys' and girls' attitudes to sport and exercise and showed how this influences their adherence to treatment plans. Evaluations of 'camps' for chronically ill children show that children experience positive changes but the most frequent theme in the evaluation was the "fun and excitement" of taking part in the "diversity of fun activities" [19].

Childhood physical activity research reveals a dearth of studies where children themselves describe *their *perspectives and understandings of physical activity. This paper reports current research which builds on an earlier qualitative study that combined focus groups, drawing and mapping techniques and photographic methods with 204 children aged 4–12 years in metropolitan and rural South Australia, asking "What are children's experiences of physical activity, play and sport?" and "What do children want to tell adults?" [20]. In that study, despite a focus on eliciting children's accounts of participating in physical activity, there was no mention by children of chronic disease as a barrier. In the current study physical activity included physically active play and activities both at school and 'home' (backyard, playground), organised sports with clubs or schools or recreational play (hobbies) such as dancing, judo, orienteering or canoeing.

The current study aimed to increase our understanding of physical activity among children with a chronic disease and promote children's participation in research concerning their health. This exploratory study aimed to discover:

• How South Australian children living with asthma, type 1 diabetes or cystic fibrosis describe their experiences and perceptions of physical activity, exercise, sport and play.

• How these children view physical activity's barriers and enablers.

• The perceptions of parents of children living with asthma, type 1 diabetes or cystic fibrosis about their children's participation in physical activity, exercise, sport and play.

## Methods

### Theoretical Frameworks

This research concerns a new area of inquiry that sought an understanding of how children living with asthma, type 1 diabetes or cystic fibrosis describe and understand their experiences of physical activity. Coherent theoretical frameworks informed this study. Interpretive phenomenology guided the methodological approaches. The 'new paradigm sociology of childhood' [21] provided the imperative of attending to children's accounts and ensuring that they have a direct voice in the production of child health research. This approach theorises children's agency and embodies Article 12 of the UN Convention on the Rights of the Child (1989) which respects a child's right 'to say what they think about matters that affect them and to have those views taken seriously' [22].

The qualitative methodology allowed for exploration and interpretation of the ways in which children and young people with a chronic illness and their parents described their experiences of participating in physical activity. This approach was based on both interpretive phenomenology and data collection approaches consistent with enabling the active participation of children and young people. As a qualitative research approach, interpretive phenomenology aims to offer a plausible interpretation and detailed description of a phenomenon that reveals, deepens or extends our understandings of human experience as lived and articulated by study participants. Phenomenology has both descriptive and interpretive strands within its wider tradition. While these are not mutually exclusive approaches, descriptive phenomenology draws more from Husserlian philosophy and interpretive phenomenology owes more to Heidegger's work [23]. Interpretive phenomenology from this tradition "focuses on the person in context, on commonalities of language, practices, everyday shared understandings and ontological questions concerning persons' being-in-the-world" (p 190) [24].

### Ethics Approval

The study was conducted in accordance with the "Statement on Human Experimentation" by the National Health and Medical Research Council of Australia, and with the Helsinki Declaration of 1964, as revised in 1983 [25] It was approved by the following Human Research Ethics Committees: Children, Youth and Women's Health Service, Flinders University, the University of South Australia and The Department of Education and Children's Services.

### Sampling and Recruitment

Purposeful sampling was used to ensure information-rich cases that resulted in an in-depth understanding of the research question rather than empirical generalisations [22]. For the study we narrowed the spectrum of chronic illnesses to children and young people with type 1 diabetes, asthma or cystic fibrosis. The selection of children with these three diseases was in response to three main factors. First, we responded to assessors' helpful comments received in response to an earlier grant proposal who felt that our initial sample frame of 'any chronic childhood illness' was too broad and that we should focus on two or three of the more common conditions. Second, asthma and type 1 diabetes are common and serious childhood illnesses whose incidence and prevalence are increasing [26]. Third, we responded to concerns raised by our industry partner in the study, the SA Dept of Education & Children's Services, whose staff are involved in devising and using school health plans for such children and who had specific concerns and questions related to cystic fibrosis and children's physical activity that our research could help answer.

Children and young people with a chronic illness and their parents were recruited through a large tertiary paediatric hospital's Diabetes Clinic and Respiratory Clinic. Potential participants were sent or given an Information Pack explaining the study (in both adult and child-friendly versions) via the Diabetes or Respiratory Specialist Nurse. Potential participants had the opportunity to read the information at home and then voluntarily make contact with the research team to ask any further questions and/or consent to participate. The researchers did not know the names of families who received information packs and the relevant clinical team did not know who participated in the study. Written consent was required from the child's parent(s) and children and young people had an assent clause that they could sign to indicate their agreement to participate in the study. In the first stage of data collection 25 children and young people participated. Fourteen had a diagnosis of type 1 diabetes, 6 had a diagnosis of asthma or chronic respiratory illness and 5 had a diagnosis of cystic fibrosis. The children's ages ranged from 4–16 years old with an average age of 9.5 years (see Table 1). As the study was in collaboration with the South Australian Department of Education and Children's Services, the children and young people recruited were all school aged (some schools include pre-schools as part of their services). Twenty-five parents of these children were also interviewed.

**Table 1:** Stage 1 participant sample – age, gender, chronic disease

**4**	Male	1	2	1
**5**	Male			1
**6**	Male	1		
	**Female**		**1**	
**7**	Male	1		
	**Female**	**1**		
**8**	Male	1	1	
**9**	Male			1
	**Female**	**1**		
**10**	**Female**	**2**	**1**	
**12**	Male	1		
**13**	Male	1		1
	**Female**	**1**		
**14**	**Female**	**1**		
**15**	Male	2		
	**Female**		**1**	
**16**	**Female**			**1**
**Total**		**14**	**6**	**5**

Participation in Stage 1 of the study involved a combination of focus group interviews, drawing maps, taking photos and designing 'traffic light' posters (see Figures 2 and 3) by the children and young people. Using multiple data collection methods within a framework of children's active participation is consistent with global calls for participation and involvement of children and young people in health research and practice [27]. This also helped engage and interest children, leading to the collection of more diverse and valuable data.

### Focus Groups

Focus groups were held separately with children and young people and with their parents. With the participants' consent interviews were audiotaped. Focus groups are a data collection method appropriate for research that seeks to explore a range of people's opinions, experiences and feelings, in a group, without any goal of conformity or consensus [28]. This method is also recommended for children to capture their perspectives, original ideas and experiences [29]. Group interviewing is a research technique that seeks advantage from the group dynamics that can be a stimulus to elaboration and expression by participants [30].

Between 4–6 children participated in each focus group. Where possible, we tried to hold groups involving children of approximately similar age ranges. The groups were usually of 30 minutes duration and facilitated by two members of the research team with one observing and taking written notes. Generative questions phrased in child-friendly ways explored such issues as:

• How do the children describe the physical activities that they take part in at school?

• Are they able to access and take part in the activities that they would like to?

• How do they balance and manage physical activity within the context of their illness?

• How do children think that parents and teachers influence their physical activity?

• What do the children see as the barriers and enablers to their physical activity?

Parent focus groups consisted of 4–6 participants, were held separately from the children's groups and lasted between 30–60 minutes. Questions for parents included:

• How do you see the place of physical activity in your child's life?

• What would you say are your child's favourite physical activities? Why do you think these are their favourites?

• What do you think is the school's role in planning and providing a physical activity program for your child?

### Mapping

As part the focus group the children and young people were invited to draw a map of their physical activities including where they took place. Children and young people often annotated their drawings spontaneously or one of the researchers assisted younger children with their words or sentences. All participants were encouraged to explain their map to one of the researchers and the researcher kept separate notes. MacDougall, Schiller and Darbyshire [20] found that drawing and mapping elicited data and information that was distinct from that gained through focus group discussions. Mapping is regarded as especially suitable for younger children as they begin to explore spatial location and mental mapping of their first hand experiences [31]. For a more detailed discussion and critique see Darbyshire et al [32], Punch [33] and Prosser and Schartz [34].

### Photovoice

'Photovoice' was first developed by Wang and Burris [35] as a form of 'visual sociology'. Encouraging children to take photographs helps them visualise and record their physical activity patterns and contexts. Cameras can be empowering for children as they choose what to photograph and how to annotate the photographs. Each of the focus group children was given a disposable camera and asked to take 'Me and my activities' photographs over a two-week period, with adult help if necessary. As a visual data production strategy this enables children to depict people, places and activities that are important to them. However, it is essential to ensure that the children are able to participate in discussing and interpreting their photos [32]. Thus within a month we met with the children again in small groups to enable them to create 'traffic light' posters using their photos (Figures 2 and 3). One researcher explained at the commencement of the group that the green circle was to record activities they found 'easy' the amber circle for activities they found 'a little more difficult' and the red circle for activities they found 'hard'. The researchers spent time with each child as they explained the significance of each photo and listened to the child's rationale of how they classified each photo. The children were encouraged to annotate their photographs and posters. The researchers kept written notes of the conversations. Following each group the posters were discussed individually and collectively by the researchers, scanned for data storage and the original poster was laminated and returned to the child.

### Stage 1 – Data analysis and interpretation

Data analysis and interpretation occurred simultaneously throughout the data collection stage. The research team met after each session of data collection and our discussions and debates were informed by a wide range of professional perspectives, including physiotherapy, children's nursing, psychology, public health and early childhood education. Interpretive phenomenology does not claim to 'bracket out' researcher experience but rather acknowledges the biography and background understanding that the researcher brings to interpretation and understanding. Having at least two of the research team present during data collection and analyses sessions provided a robust check against one person's perspective having an unchallenged interpretive influence.

Each focus group interview was transcribed verbatim and any names or identifying data replaced with pseudonyms. Using QSR N-Vivo software, each interview was read and coded [36]. The codes were then clustered into themes and sorted according to the categories of chronic illness and age groups to identify any themes specific for each group and the themes that were common across all groups. In addition data sets (Table 2) were created for each child that linked their maps, interview and poster with the parent interview.

**Table 2:** Example of 3 data set summaries

**Data Set**	**Alice – 10 year old girl with Type 1 Diabetes**	**Tom – 5 year old boy with Cystic Fibrosis**	**Natalie – 16 year old girl with Asthma (from rural area)**
**Map Illustrations**	Drawings of herself skipping with a rope and another playing netball	Drawings of playground equipment and running with his friends	Drawings of her house with a bike, cricket pitch and golf course close by and her school with basketball court, gym, cricket nets and dance studio.
**Poster Content**	Green (easy) -photos of herself playing netball and playing with her dog Orange (a little more difficult) -photos playing ball games with friends, riding a scooter, relay running for school team Red (hard) – written phrase 'Life's not that hard' and pictures of jelly beans 'in case I get low'	Green (easy) – photos with his brother playing at park, jumping on trampoline, riding his bike, bouncing & kicking balls. Orange (a little more difficult) – a picture climbing a tree. Red (hard) – photos playing at the beach and holding a rabbit in the backyard.	Green (easy) photos playing cricket, golf and dancing. No orange Red (hard) – Photos of outdoor sports fields and she has written the caption that she 'can't play outdoor sports in winter'.
**Focus group and commentary re map/poster**	Alice reports at home she plays outside with her dog, shoots goals or jumps on her trampoline. At school she skips with her friends and plays softball and netball for the school team. Jelly beans are kept in school office in case she feels 'low' or requires them before playing a game that needs lots of energy.	Tom says he plays with his friends at school and they run and play on equipment. He also kicks the ball with them and says he can do everything they can. Tom says he gets very tired playing in the sun and plays in the shade. In reference to his poster he says climbing a tree is hard as "you may fall down" and "Mum says I get tired at the beach" and holding a pet rabbit is hard because they run away.	Natalie says she loves playing cricket and golf. She tap dances 5 times a week and studies dancing as a school subject. She reports she usually takes her 'puffer' before sports. She participates in sports at school but had one bad experience with a teacher who would "scream" at her if she sat down and "she made us (other class members as well) keep going when you just couldn't".
**Parent's focus group/interview**	Her mother describes Alice as active and she is more 'sporty' than her brother. She encourages Alice to play sport especially netball but makes sure she eats correctly and has 'back-up' carbohydrates. She reports the school is supportive and teachers give Alice a sweet before running activities.	Tom's mum says he is very active playing but is too young for organized sport. She reports he plays a lot with his brother (a bit older) and they run, play ball games and play wrestle. She reports Tom gets tired after school and the sun can make him tired as well.	Natalie's parents reported they were not very active but Natalie was very active. They drive her long distances for dancing (350 kms each week) but are pleased she loves dancing because 'it is dry and warm'. They report Natalie needs Ventolin when she plays sport but can still get puffed and they have had problems with some teachers not accepting her capacity and limitations when she is not feeling 100%.

Emerging interpretations of data were then discussed, debated and questioned in analysis meetings of the full research team, and in meetings with the Project Advisory Group consisting of the type 1 diabetes and Respiratory Specialist Nurses and the Manager of Children's Health and Education Support Services within the Department of Education and Children's Services. The rationale for using the three different methods for data collection was to triangulate and to obtain the richest possible data, that is, focus groups (verbal responses), mapping (graphic responses), and photovoice (personal and creative/expressive response) for each child.

### Stage 2 – New sample for checking and validating

A possible limitation to Stage 1 of the study was that the study topic may have attracted a biased sample of families who were physically active as opposed to those who viewed physical activity as a lesser focus or priority in their family lives. From our initial data analysis the Project Advisory Group raised concerns that our sample may have been biased towards families who self-selected on the basis of their active participation in physical activity. The researchers then decided to sample from a wider group of children and young people with either asthma, type 1 diabetes or cystic fibrosis to invite them to comment on and discuss the findings from Stage 1 and to add their own perspectives and experiences. These students were recruited through four schools where relationships had been established through a previous study. Parents of children with type 1 diabetes, asthma or cystic fibrosis within these schools were sent an Information Pack and Consent Forms for consideration. These were sent directly from the school and the names of families were unknown to the researchers unless a positive response was received. Following parental consent and child assent a focus group was conducted in each of the four schools involving a total of 12 students aged between 5–12 years old. Of these students 8 had asthma, 2 had type 1 diabetes and 2 had other chronic illnesses. The focus groups started with a general discussion using the same format as Stage 1 of the study. The researchers then asked open-ended questions that related to previous findings to seek the children's views and perspectives.

The feedback from each of the children's 'consultancy' focus group was recorded, transcribed and coded. Themes were then compared and contrasted with the themes from Stage 1 of the study. The second sample of children provided data very similar to the participants in Stage 1. This result suggested that the initial sample of children were not as 'biased' or atypical as was suspected and strengthened confidence in Stage 1 data interpretation.

All names used in this paper are pseudonyms.

## Results

Children and young people who participated in this study described their participation in a wide range of physical activities including both organised sport and play at school and home. Their parents' accounts supported these findings. The data sets illustrated the consistency of findings (Table 2). The data from each child's or young person's maps, posters and interviews was closely linked and concurred, for the most part, with the data from parent interviews. Both groups demonstrated strong positive beliefs that participation in physical activity did not need to be hindered by a chronic disease. From these finding two overarching themes emerged:

• Children and young people beliefs and perceptions that they 'could do anything' that their peers did in relation to physical activity; and

• Parental beliefs that they 'would do anything' to enable this to happen.

### "I just put my mind to anything and I can do it." The children and young people's perspectives

Children and young people participating in this study rarely cited any barriers to being involved in physical activity. The title of this paper was taken from a quote that summarised a common perspective expressed by many of the participants:

There's nothing I can't really do because I just put my mind to anything and I can do it. (Martin, a 13 year old boy with type 1 diabetes)

Nadia said that only the level of sickness that required hospitalization would be a deterring factor:

It doesn't really stop me from doing stuff except for when I get really sick then I have to go to hospital. (Nadia, a 15 year old girl with asthma)

Jake (a 13 year old with cystic fibrosis) not only kept up with his siblings but:

He's probably the most active of them all. (Father of Jake, a 13 year old with cystic fibrosis))

From the children's and young people's discussions, maps and posters it was apparent that they engaged in a wide variety of activities such as organized sport, including netball, swimming, basketball, tennis, table tennis, cricket, football, gymnastics, golf, hockey, cross country, obstacle course, soccer and canoeing, in addition to play activities including playing with friends, walking the dog, bike riding, skateboarding, climbing trees, playing handball, riding a scooter, playing 'chasey' and dancing. Many children described sporting commitments such as:

I do little athletics. I do heaps of activities every Tuesday night. I also play tennis on Saturdays in juniors and sub-juniors. (Chad, a 10 year old boy with asthma)

**Figure 1:**
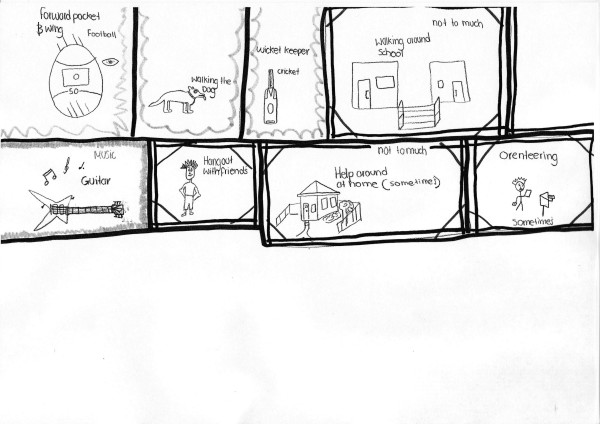
**Mark's map**.

Mark, a 15 year old with type 1 diabetes, drew a schematic diagram in Figure 1 showing a range of activities from football and cricket to hanging out with friends, music and walking the dog. Next to his drawings of helping around the house, walking around school and orienteering his annotations of *sometimes *and *not too much *suggest teenage irony, rather than symptoms preventing participation as he did not cite any restriction when depicting football and cricket, which are sports requiring significant exertion.

**Figure 2:**
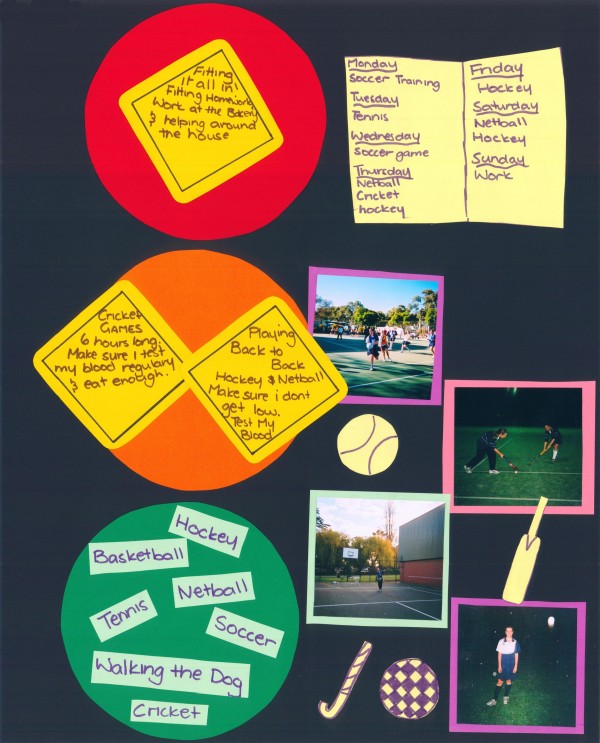
**Sophie's poster**.

Sophie, a 14 year old with type 1 diabetes, drew a schematic map listing the many activities in which she was involved and some of the places where they occurred. Her map listed netball, tennis, basketball, gym, cricket, soccer, the playground and swimming. Sophie's Poster, (Figure 2) showed a timetable of all these activities, depicting a very busy life. Involvement in multiple sports and activities was not unusual amongst the participants.

**Figure 3:**
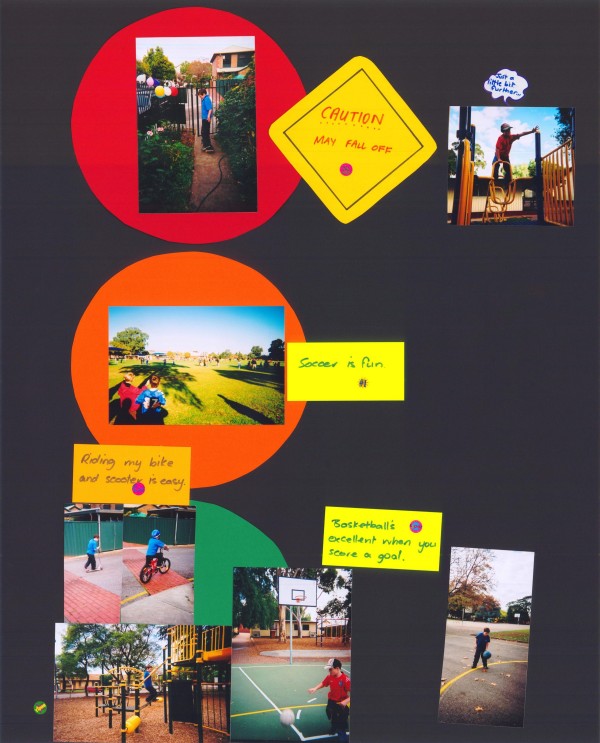
**Alexander's poster**.

Children and young people did not nominate asthma and type 1 diabetes as significant barriers to participation in physical activities, either in focus groups or in the red (or 'difficult' area) on their traffic light posters. Alexander, a 9 year old with cystic fibrosis and asthma included in his poster photos of him playing basketball and soccer, riding a bike and a scooter and these activities were all classified as 'easy' in the green section of the poster (Figure 3). Skateboarding however was in the red section as he was learning and was 'scared to go fast'. Alice, a ten year old girl with type 1 diabetes, told us *"there is nothing I can't do" *because of her type 1 diabetes and consequently, *"that's why I don't know what to do in the top one' *indicating the red circle of her poster. Activities that were assigned to the red circle by other children included a photo of football by James, an eight year old with asthma, not because of his disease but because "*It's hard to kick the ball"*.

Children and young people said they were included in sport and physical education sessions at school. They remembered clearly occasions when they felt that they were being singled out or treated differently. For example Eloise gave this account of when she was younger:

One of the PE teachers used to treat me as if I was about to die, that's so annoying. Say something like, if we were playing chasey I'd never be caught and she would just whisper in people's ears don't get 'Eloise'. So you just stand there and people run past you. (Eloise, a 10 year old with asthma)

Conversely Natalie, a 16 year old girl with cystic fibrosis and asthma, had difficulties with one physical education teacher whom she described as someone who did not understand that sometimes she needed a rest before continuing with a sporting activity. Symptoms of their illness, which may be exacerbated by certain activities, were viewed as transient disruptions that could be overcome and the activity continued. For example, children and young people with asthma or cystic fibrosis did indicate that a lot of running could make them short of breath and they needed to stop for a rest or medication before they resumed running again.

*When I feel too puffed out I just stop and rest and *[then]*run again. (Daniel, a 4 year old with asthma)*

A 'puffer' was used before or during sport but was rarely used in the playground and was generally kept in the school office. Children with asthma indicated that cold weather could trigger their asthma and one young girl indicated she played more indoor sports in winter for this reason. Winter was also a problem because "*I get coughs more in the winter and get colds really easily"*. The summer months brought problems of allergy-induced asthma and one child explained she had an allergy to grass and needed to be careful when playing on the oval. Children with cystic fibrosis could not play sports in the sun, or swim in public swimming pools because both activities posed a direct risk for managing their illness. However, despite these restrictions all the children and young people reported participating in many sports and physical games that avoided these situations.

Children and young people with type 1 diabetes indicated the need to check their blood sugar levels before playing sport but not before playground games. Ruby, a 10 year old with type 1 diabetes, has a caution sign on her poster warning "*Finger pricks before sport*" and Peter, a 15 year old with type 1 diabetes, described a more intensive regime:

Sometimes I check my own blood sugar and then during exercise I pause every half an hour to an hour to eat some extra carbohydrate food whether a muesli bar or jelly beans or something as you're running around. (Peter, a 15 year old with type 1 diabetes)

All the children with type 1 diabetes had an understanding of a 'hypo' and could describe symptoms that were indicative of a low blood sugar and what action they needed to take. Examples included:

When I'm playing soccer and my legs get weak and wobbly that's when I need to eat some 'lollies' (what Australian children call sweets or candy). (Liam, a 6 year old with type 1 diabetes)

No children said they were teased because of chronic illness. On the contrary, many mentioned special friends who accompanied them to the school office if they needed their asthma puffer or jelly beans because they were feeling low in sugar. Two parents spoke of their children not wanting their illness '*publicised*' via a diabetes information session for other children in the class or wearing a 'medic alert' bracelet, but both these children indicated it was acceptable for their best friends to know about their illness. Parents played an important role in the management of their child's illness as reported in the next section.

### "I do what I have to do so she can do anything": The parent's perspective

Parents of the children and young people involved in the study were interviewed separately from their child. Parents acknowledged the importance of physical activity for general health and wellbeing and were aware that these benefits were just as pertinent for their child with asthma, type 1 diabetes or cystic fibrosis. In addition they were able to give reasons for encouraging their child's level of physical activity to directly improve their disease management, including 'shaking his lungs up' that assisted chest physiotherapy for children with cystic fibrosis and for weight control for children with type 1 diabetes.

The group discussions with parents conveyed the depth of parents' knowledge of their child's illness, and the necessity of being vigilant in their planning and structuring of activities to maximise their child's participation in physical activity and to minimise potential complications that may arise from their illness. As summarised by the mother of Ruby (a 10 year old with type 1 diabetes), "*I do what I have to do so she can do anything." *For these parents, type 1 diabetes, asthma or cystic fibrosis was not seen as a barrier to physical activity and especially involvement in sport, however it involved parents *"playing a juggling game a bit more"*. Parents of children with cystic fibrosis indicated that the child's illness did not stop them from being involved in physical activities but it did slow them down at times and they had to learn when they needed to rest. As this parent commented:

Like in soccer I noticed after a couple of minutes he was very, very breathless. The coach doesn't know too much about CF so I was just watching him and yeah he coped and he kept wanting to play but he really needed a break. After resting a couple of minutes he is as good as gold. (Mother of Alexander a 9 year old with cystic fibrosis and asthma)

This daily management was often taken for granted as simply part of their everyday family life until, as one mother observed:

You try to have a holiday with another family – that's when you realise that you're not normal. (Mother of Martin, 13 years old with type 1 diabetes)

The mother of Alice, a 10 year old with type 1 diabetes explained that she was *'always planning'*.

Depends if she's going out to sport you know you always make sure she's eating, especially her netball, a very active sport you make sure she's eaten a proper breakfast, make sure you've got lollies and a back-up carbohydrate or you know that there's shops nearby. (Mother of Alice, 10 years old with type 1 diabetes)

Planning and vigilance were especially evident in the discussions with parents of children with type 1 diabetes, especially when the children participated in physical activity. The mother of Ruby (a 10 year old with type 1 diabetes) explained that when Ruby undertakes activities that will require sustained energy (including bike riding as a family or cross country running at school) she needs to ensure she has extra food before she leaves home and that Ruby carries a 'backpack' with food she can eat during the exercise. Liam (a 6 year old with type 1 diabetes) is given the responsibility for preparing the food for the 'hypo box' for school but his mother feels that she needs to check it afterwards due the serious consequences if food is not available to him and he feels his blood sugar is low. Liam takes the box to all sports lessons and to after school care. David (a 12 year old with type 1 diabetes) can now administer his own insulin but his parents always check the dose first as *"it could be a matter of life or death with the wrong dose"*. This year his parents were happy for him to go on a school camp without one of them present, but he would telephone them each night before he administered his insulin so they could check the amount of insulin he had drawn up according to his blood sugar levels. David's parents said they tried to balance the need to be vigilant while avoiding stress for their son by conveying their anxiety:

The more we [parents] stress about it the more he [son] stresses about it and his blood sugar goes higher. (Mother of David, 12 years old with Type 1 diabetes)

Such detailed planning and use of contingency strategies did result in parents feeling reluctant to transfer this responsibility to others, including parents of their child's friends. It was difficult for these parents to allow their child to be taken to and from sport by other families *'just in case' *a problem arose. The mother of Oliver (a 7 year old with type 1 diabetes) explained:

*So I guess the long and the short of it is that's limited, limiting in that you can't be flexible, I don't feel at this point in his life I could just let him be taken by families to sport, you know people could be talking or anything could be happening on the field. I'm there for him on all those sporting activities but I just have to try to manage that with work and the days because I work obviously to five o'clock and that sort of thing*.

Parents of children with type 1 diabetes explained the importance of their child being supervised by an adult when swimming in case of a hypoglycaemic episode in the pool. The mother of Mary (a 9 year old with type 1 diabetes) always goes with her to the pool even when her friend's parents are also present. When other parents in the group raised the issue of other parents being temporarily responsible for their child Sophie's mother described the level of responsibility she expected from other parents:

Basically she [Sophie] looks after herself, if she does anything really strange I tell them call an ambulance. (Mother of Sophie, 14 year old with Type 1 diabetes)

There were examples of parents using 'background strategies' to manage their child's type 1 diabetes without the child being aware. Mary's mother explained how she manages her daughter's blood sugar levels and energy expenditure on a school morning:

We walk to school in the mornings and that often means that she can have a bigger breakfast than she might otherwise have and I don't have to worry about it because I know we're going to spend that extra energy walking to school rather than taking the car. If she's woken up not very hungry I don't stress too much if she doesn't eat very much I just say we'll drive to school today. (Mother of Mary a 9 year old with type 1 diabetes)

Other parents highlighted the importance of their child learning to self-manage and encouraging them to monitor their own blood sugar levels, administer their insulin and decide what carbohydrates they should eat. However most parents explained that they did still keep *'a close watch' *over all these tasks although their child may not be aware of this. Several parents confided that when their children were away they sometimes made contact with the adults to check their child was 'okay' but asked that this was not mentioned to the child.

Parents of children with asthma also described the need to plan ahead. Parents of children with asthma/cystic fibrosis ensured a 'puffer' was available for their child's use at school. Zac, a 6 year old with asthma, whose father worked from home, would ask the teachers to contact his father if he needed his 'puffer' and he would visit the school and administer the medication. Other children kept the puffer in the school office, whereas Eloise (a 10 year old with asthma) carried her 'puffer' with her at all times. The mother of Luke explains:

He thinks he can do everything which I think is great and I don't want to hold him back from activities but sometimes he has to just take five minutes beforehand to have his Ventolin to make sure his airways are nice and opened. (Mother of Luke a 4 year old with asthma)

Often the manner in which parents managed their child's capabilities in relation to their chronic illness was subtle and the child would not be aware that compromises had been made. Examples included parents who helped their child choose to participate in sports that required less running (for example karate). Daniel a 4 year old with asthma told us "*If I start to cough I stop and have some water*". His mother later told us that she advised him to stop for some water if he was coughing or breathless. This was a strategy to get him to stop and be able to rest long enough to '*catch his breath*'. The drink of water in itself was not therapeutic, it was the rest that he needed.

All parents indicated that they wanted their children to participate in all aspects of life including sport. Parents often described the efforts that they and the family would make to ensure that the child with asthma, type 1 diabetes or cystic fibrosis was able to take part in sports, play and activities and to do all of things that 'other children' do, arguably beyond what a parent of a child without a chronic illness might do. The parents of Natalie (a 16 year old with cystic fibrosis and asthma) enabled her love for dancing by driving to the dance class in another rural town involving an hour round trip five nights a week, totalling 350 kilometres each week. The travelling was felt to be worthwhile in order to enable Natalie's participation in exercise. As her mother explains "*we are relieved she loves dancing so much because it is something she can do all year round, it's indoors, dry and warm*." Others spoke of the high cost of some activities being worth it to allow their child to participate and to develop independence. Martin's mother reported that Martin wants to be like every other kid. She cited the example of where he went canoeing with his insulin pump, which became wet and 'died'. Although the pump was worth $6,000, she thought that it was more important that he canoe with his friends than save $6,000. She explained her reasoning:

I said to him, if he loses it, if he mucks it up he hasn't got it and he knows the freedom that that gives him, he knows how much that means to his treatment, it's his choice now. (Mother of Martin, 13 years old with type 1 diabetes)

Eloise (a 10 year old with asthma) was frequently driven 40 minutes each way to allow her to go horse riding. Eloise is allergic to horse hair and has only managed to ride a horse once without an asthma attack but her mother expressed she was happy for her to keep going *"because she wants to do it so much"*.

This parental attention appeared to be driven by their desire for their child to be *'as normal as possible' *and for parents their child participating in sport not only had health benefits but *'being normal was participating in sport'*.

## Discussion

### Children and young people's positive approach to physical activity

The message from the children and young people in this study was a positive one. Through their drawings, photos and words they described their involvement in a wide range of physical activities, games and sports both in and out of school. Their responses focused on what they 'can do' and they often made references to playing with friends or sibilings, and in some instances made references to being more active than their peers.

Our study findings were contrary to the qualitative study by Rhee et al [37] where adolescents (ages 12–18) reported their involvement in sport was often less than their peers. Rhee et al [37] also found young people with asthma described feelings of 'missing out' in relation to physical activity, yet the young people with asthma in our study (ages 4–16) reported that asthma did not stop them from participating but it could make some activities harder. Possibly the more negative response from Rhee et al's study participants may have been triggered by their focus group question "What kind of things does asthma stop you doing?" whereas our study asked questions that were more positively framed for example "What physical activities do you like doing?" Can you do all the activities you would like to do?" and "Does having [disease name] make it harder?"

It was also evident that the children and young people in our study enjoyed physical activity. Lang et al [14] found children with asthma who enjoyed physical activity were more likely to be active. Badlan's [38] study of the experiences of young people with cystic fibrosis found they preferred participating in exercise-based activity than physiotherapy as it was a more sociable and enjoyable way to improve their health.

Overall the children and young people in our study expressed positive beliefs in relation to participating in physical activity and they did not view their chronic disease as a barrier. Comments such as "I just put my mind to anything and I can do it" convey the importance of a positive, 'can do' attitude towards participating in physical activity. Medically focused literature supports that if symptoms of a chronic disease are properly managed having a chronic disease should, ostensibly, be no barrier to physical activity. For example Worsnop [39] writes "asthma should not be a barrier to exercise" (p. 422) and if exercise is being restricted due to asthmatic symptoms then asthma management should be improved as opposed to restricting physical activities.

Negative emotions in children with asthma combined with physical exertion were found by Reitveld et al [40] to result in children and young people (ages 7–18 years) interpreting symptoms of normal physical exertion as symptoms of asthma. The perception of asthma induced breathlessness could possibly lead to unnecessary modifications or opting out of physical activity when the root problem was a general lack of fitness. These self-reports of asthma symptoms, in the absence of objective medical evidence, illustrate the possible role of psychological factors as a barrier to participating in physical activity. The positive perceptions and beliefs in relation to physical activity that were conveyed by children and young people in our study were also echoed by their parents.

### Parental beliefs about the importance of physical activity

Parents in our study actively encouraged their children to participate in physical activities and competitive sports not only for the health benefits of physical activity but because of their belief in the importance of fostering normality by being physically active with other children. An important determinant of children's participation in physical fitness are the parental attitudes that can influence the child's belief in their own ability to participate in physical activity [41]. This finding is supported by Lang et al's [14] study where children with asthma were found to be more active if their parents believed exercise could improve their child's asthma.

In addition children with asthma who engage in regular physical activity have been found to assume better control of their illness [42]. Even if a child has exercise-induced asthma, or breathlessness due to cystic fibrosis these symptoms could be controlled by preventative medication or by having small rest breaks. Parents in our study described both these strategies and also helped their children with type 1 diabetes to manage their blood glucose levels to enable participation in sport. Alternatively more appropriate sports or activities were encouraged and supported and often the child was unaware of parental guidance and influences on their choices.

In a qualitative study of young people with asthma and their carers, Callery et al [44] found that carers sought to minimise the impact of asthma and often accepted a level of symptoms as 'tolerable'. Callery et al [44] also found carers engaged in a level of 'trial and error' in relation to treatments and activities. This was evident in some of our parents' stories with the more extreme example being the child who went horse riding when they had a known allergy to horses. However in our study this enabling culture adopted by parents was practised concurrently with diligent background planning and management strategies to minimise the risk of symptom exacerbation and maximise participation in physical activity.

### The importance of background planning

Our study highlights the important role parents play in enabling their children to participate in a range of physical activities. Parents told us that to enable their children to participate in a range of physical activities that minimised the risks of exacerbating their illness, they needed to engage in background planning. Their desire for their children to participate in the same activities as children without a chronic illness (including peers and siblings) was the driving force behind their diligent planning and support.

An example of how parental planning enabled their children to participate 'as normal' in all school activities was school camps. Parents usually accompanied their child on camps when they were younger as one of the 'parent helpers' but as the child became older it was not the norm for parents to go on camps and parents then needed to transfer responsibility to the child and teacher. Most parents felt comfortable with this arrangement as long as they were contactable and knew that their child or teacher would contact them with any concerns. Others were able to assume an 'indirect' supervisory role by asking their child to phone each night with the blood glucose reading before administering their own insulin. If parents were required to assist with chest physiotherapy they ensured that it occurred at times that were least likely to interrupt 'normal' activities.

### Passing on responsibility to other adults

The level of responsibility felt in relation to the importance of background planning, and the need for continual monitoring of their child's health status, resulted in parents often being reluctant to give other parents the responsibility of looking after their children in relation to sport and physical activity. This was most evident for parents with a child with type 1 diabetes. Parents of children with type 1 diabetes described their constant planning and managing of their child's food intake, energy expenditure and monitoring of blood glucose levels. This is similar to the theme of 'constant vigilance' reported by Sullivan-Bolyai et al [45,46] for the day-to-day care performed by mothers of young children with type 1 diabetes. Parents were very aware of the potential consequences of hypoglycaemia in relation to exercise, reinforcing Wolfsdorf's [9] findings about the need for close monitoring of the child with type 1 diabetes undertaking physical activity, and the recommendation for proper guidance and preparation to prevent hypoglycaemia during and after exercise. Feeling unable to ask other parents to accompany their child to activities placed a heavy a burden on this group of parents and reduced the opportunities for them to share the duties of accompanying children to sport. One parent had decided not to work to ensure that she could always be available to assist with her child's activities. Although all parents of children assist and plan for their children's activities we found that for parents of children with type 1 diabetes, asthma and cystic fibrosis the potentially grave consequences of poor disease management added a significant layer of responsibility. This level of commitment shown by parents could potentially be a barrier to children with chronic illness participating in sport where families lack the necessary resources.

### Parental planning becomes self-management

The high levels of parental planning and involvement to ensure their child with type 1 diabetes did not experience hypoglycaemia or that their child with asthma or cystic fibrosis did not experience acute bronchospasm or severe breathing difficulties raises the issue of when and how does a child develop self-management skills. Although the children and to a large extent adolescents, seemed oblivious to this level of planning and intervention, young people and children described scenarios of having their 'puffer' available when they played sport and taking short breaks (often when coughing) to allow their symptoms to settle but then continuing with the activity. Children and young people with type 1 diabetes described needing to take their blood sugar levels before and sometimes during sport and the need to eat before exercise and take emergency sugar foods. This balance between parental involvement and child self-management for type 1 diabetes accords with studies that describe the norm of parents taking a dominant managerial role until around age 11 and a transition period of child management with parental checking until age 15 [47,48]. Even the young children with asthma described activities of self-monitoring (using a 'puffer', stopping to rest) which Burkhart and Ward [49] recognise as the foundation of self-management for asthma. However the level of parental planning in this study appeared to be the foundation for their child undertaking physical activity as a 'normal' lifestyle behaviour and believing that *'they could do anything'*.

### 'Normalisation' of physical activity

The results of our study provided accounts of children's, young people's and parent's experiences that illustrated how they were not only successfully managing a chronic disease to maximise participation in physical activity but they were also maintaining a sense of family 'ordinariness'[50]. Chronic disease was not framed in terms of a barrier. If certain sports or activities were contraindicated for their disease they chose other sports. If symptoms of the disease were exacerbated by exertion then strategies were implemented to allow continuation of the activity. These families emphasised their capacity to overcome potential barriers to participation in physical activity and to be seen as 'normal'. 'Normalisation' or 'ordinariness' from this viewpoint is a form of coping and treating the disease and treatment regime as being part of a normal family life [51]. Even when they went away with other families and the differences in their 'normal' family life were more evident strategies were put in place to ensure treatment regimes continued as 'normal' so their child could participate with other children. Similar to the findings of a qualitative study of children with a chronic blood disorder [52] parental support in our study offered their children considerable protection from both the practical problems of the disease but also the potential negative views and actions of others.

### Strengths and Limitations

The interpretive approach of this study allowed insight into how children, young people with asthma, cystic fibrosis or type 1 diabetes, and their parents, perceived physical activity. By describing the actions they took to ensure chronic illness caused minimal disruption we have illuminated the perceptions and beliefs of our study participants in relation to the importance of physical activity for health and social reasons. However the findings are from a small scale study in one state of Australia and we would caution against any overgeneralisation.

## Conclusion

The message of physical activity being important for all children and young people, including those with a chronic illness, is exemplified in our study. We found none of the "victimization" or "bullying" of children with a chronic disease by teachers or peers described by Peters et al [53]. We found that parents are taking a very active role to enable their child's participation in physical activity. Further research into the modes and dynamics of parental interventions and to what extent they enhance a child's participation in physical activity would be valuable.

In our study a positive attitude towards physical activity was found to be an important motivating factor for participation. We appreciate that tangible resources are often required in order to translate attitudes into actions and that for some disadvantaged families, enabling various forms of physical activity for their child may pose a real challenge. This raises the future research question of how socio-economic status impacts upon parents' ability to enable and support their child's physical activity. Parents in this study described driving long distances for their children to participate in activities, some of the sporting activities described would require equipment and sporting fees, and parents' appeared to be willing to take extraordinary measures to ensure their child's participation, even risking a $6,000 insulin pump. This parental investment of time and money to enable their child to participate in a range of sporting activities raises the question of potential health inequities when it comes to resource availability for physical activity opportunities as part of any equitable, preventative health strategy. The importance of physical activity programs within schools and community programs that provide opportunities for sport with minimal financial cost to families should therefore be supported by government policy to ensure that all children and young people, including those with a chronic illness, have access and opportunity to participate in a wide range of activities.

## Competing interests

The authors declare that they have no competing interests.

## Authors' contributions

JF prepared the manuscript and participated in data collection and analysis; CM, PD, WS designed the study, participated in data collection and analysis and revisions of the manuscript; MS managed the study including data collection and analysis and participated in revising the manuscript. All authors gave final approval of the version to be published.

## Pre-publication history

The pre-publication history for this paper can be accessed here:


